# Solid Serous Cystadenoma Mimicking Neuroendocrine Tumor of the Pancreas: A Case Report

**DOI:** 10.1155/crgm/4945872

**Published:** 2025-09-02

**Authors:** Adriano Carneiro da Costa, Jayant Kumar, Mohamed Ali Chaouch, Isabella Reccia, Camila Ramos Martins, Flavio Silano, Paulo Cezar Galvão do Amaral, Nagy Habib

**Affiliations:** ^1^Department of Surgery, Hospital of Clinics, Federal University of Pernambuco, Recife, Brazil; ^2^Department of Surgery and Cancer, Hammersmith Hospital, Imperial College London, London, UK; ^3^Department of Visceral and Digestive Surgery, Monastir University Hospital, Monastir, Tunisia; ^4^Department of Surgery of the Upper Digestive System, São Rafael Hospital, Salvador, Bahia, Brazil

**Keywords:** laparoscopic pancreaticoduodenectomy, pancreas, pancreatic cysts, solid-type serous cystadenoma

## Abstract

Solid-type serous cystadenoma (SSCA) of the pancreas is an extremely rare benign condition among pancreatic cystic neoplasms. Although the imaging characteristics are not pathognomonic, this entity may mimic other solid pancreatic tumors. In particular, it can closely resemble a pancreatic neuroendocrine neoplasm (pNEN) on radiologic studies. We report the case of a 67-year-old woman who presented with abdominal pain. Preoperative abdominal magnetic resonance imaging (MRI) with contrast demonstrated a solid, hypervascular lesion at the pancreas, measuring 3.5 × 2.2 × 1.9 cm, with marked enhancement on dynamic imaging. A nonfunctioning pancreatic neuroendocrine tumor was initially suspected. The patient underwent laparoscopic pancreaticoduodenectomy. Histopathologic examination revealed a solid variant of serous cystadenoma, a rare subtype of pancreatic cysts. The patient remains asymptomatic, with no evidence of recurrence or residual disease at 7 years postoperatively. This case highlights the diagnostic challenge of distinguishing solid-type serous cystadenomas from other pancreatic lesions based on imaging alone, underscoring the role of pathology in definitive diagnosis.

## 1. Introduction

Serous cystic neoplasms (SCN) are rare cystic tumors of the pancreas, representing less than 1% of all primary pancreatic lesions [1, 2]. SCN is currently categorized into five subtypes: serous microcystic adenomas, serous oligocystic ill-demarcated adenomas, solid-type serous cystadenomas or solid serous adenoma, von Hippel–Lindau disease-associated cystic neoplasms and serous cystadenocarcinomas [3, 4]. Among them, solid-type serous cystadenoma (SSCA) is by far the rarest subtype and has been reported in the literature only in 15 cases [3].

A large proportion of pancreatic cystic neoplasms are now being detected due to recent improvements in imaging modalities, which result in greater accuracy in the incidental detection of small tumors in the pancreas [3, 5]. However, accurately diagnosing solid variant of serous cystadenoma before surgery is challenging due to its rarity and similarity in imaging findings to pancreatic neuroendocrine tumor [3].

SCN is a basically benign entity, so watchful observation is recommended unless the patients appear to have symptoms, or the tumor turns to have malignant potential [3, 6]. However, correctly diagnosing SSCA preoperatively remains a challenge and may be misidentified as other pancreatic cystic neoplasms or solid tumors, especially when they are small or contains intratumoral hemorrhage that imparts a more solid appearance [7]. In the current study, we report a case of SSCA which was preoperatively diagnosed as pNEN and underwent laparoscopic pancreaticoduodenectomy.

## 2. Methods

### 2.1. Case Presentation

**Figure 1:**
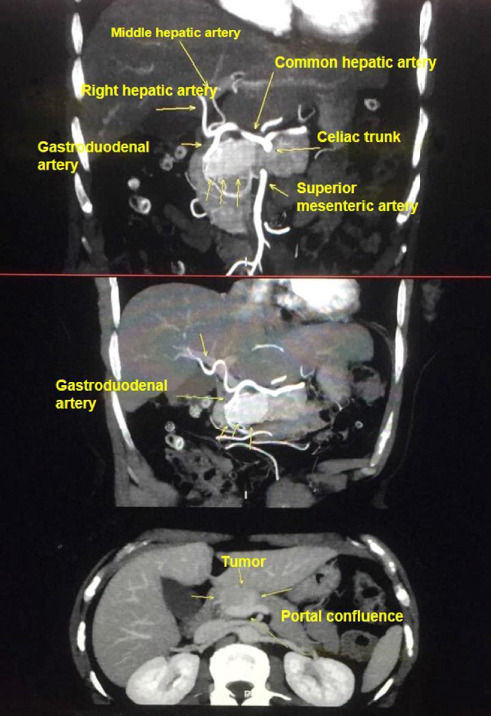
Contrast-enhanced magnetic resonance imaging, showing a 3.5 × 2.2 × 1.9 cm hypervascular solid mass at the head and body of the pancreas.

**Figure 2:**
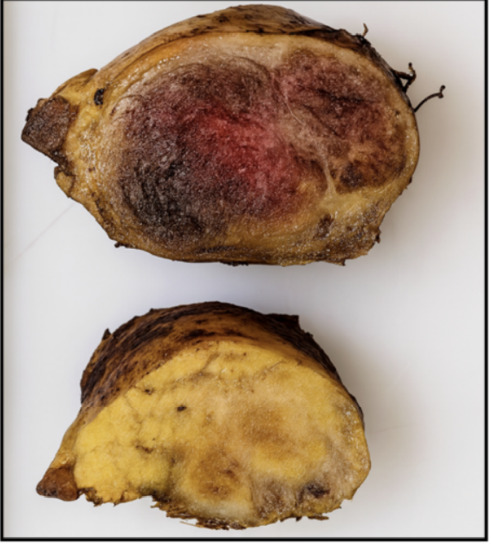
Gross specimen of the pancreatic head demonstrating a well-demarcated, solid tumor measuring 3.0 × 2.2 cm.

**Figure 3:**
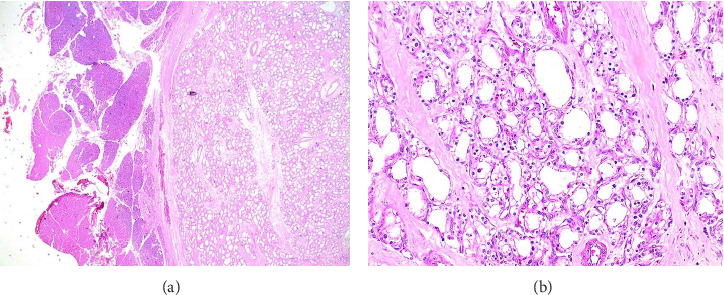
Postoperative pathological examination revealed a solid variant of serous cystadenoma of the pancreas. (a) Full section view (HE stains, 40x) showing well-circumscribed tumor capsulated by thick fibrosis. (b) High magnification (HE staining, ×400). The edge of the tumor contains densely packed microcysts, organized as a single layer of cuboidal epithelial cells with shiny cytoplasm. There is no necrosis, cytological atypia, or abnormal mitotic figures.

A 67-year-old woman presented with significant abdominal pain. Clinical abdominal examination was unremarkable. Laboratory data were within the normal range, including serum markers of pancreatic tumors (carcinoembryonic antigen [CEA], carbohydrate antigen 19-9 [CA19-9], and chromogranin A [CgA]). Preoperative abdominal magnetic resonance imaging (MRI) with contrast demonstrated a solid mass with low intensity on T1-weighted image (T1WI) and high intensity on T2-weighted image (T2WI) and diffusion-weighted image (DWI), measuring 3.5 × 2.2 × 1.9 cm in diameter and showed a marked enhancement of the tumor located at the head of the pancreas (Figure 1). Somatostatin receptor imaging and endoscopic ultrasonography with fine needle aspiration or biopsy (EUS-FNA/FNB) were not performed due to the radiological characteristics of the lesion and the clinical presentation at the time of diagnosis. The imaging findings were highly suggestive of a resectable solid tumor, with no features indicating unresectable. Furthermore, the patient presented with significant and progressive abdominal pain, which supported the decision to proceed directly to surgical intervention without delay. Given the radiological suspicion of pNEN, and taking into account the tumor's size, location, surgical resectability, and the presence of significant abdominal pain, the patient was referred for surgical resection. A laparoscopic pancreaticoduodenectomy (Whipple procedure) was performed. The patient had an uneventful postoperative course and was discharged on the seventh postoperative day. Histopathologic examination of the surgical specimen revealed a well-demarcated, solid neoplastic lesion measuring 3.5 × 2.2 cm located in the pancreatic head, as observed on gross examination (Figure 2). The cut surface displayed a soft, tan-whitish tissue architecture, and all surgical resection margins were free of tumor involvement (Figure 2). Microscopically, the lesion consisted of a solid, well-circumscribed tumor composed of clustered microcysts lined by a single layer of cuboidal epithelial cells with abundant eosinophilic cytoplasm (Figure 3). These findings were compatible with a solid variant of SCA. The patient had no recurrence or residual lesion evident at follow-up at 7 years.

## 3. Discussion

To date, only 15 cases of solid serous cystadenoma have been reported in the literature. We conducted a focused search of PubMed, Scopus, and Embase databases using the terms: “solid serous cystadenoma,” “solid variant serous cystadenoma,” and “solid pancreatic neoplasm,” covering all records up to January 15, 2025. After screening titles and abstracts, 15 relevant case reports were identified [3, 4, 6–13].

A summary of those previously published cases is provided in Table 1, including patient age, sex, imaging characteristics, location, treatment, and outcome. Among them, most patients were female, with tumor size ranging from 1.5 to 5.0 cm, often misdiagnosed preoperatively as pNEN or other solid neoplasms. Imaging features were frequently hypervascular and well-circumscribed, contributing to the diagnostic dilemma.

Solid serous adenoma of the pancreas was first described in 1996 [14], being an extremely rare benign tumor of the pancreas and has been reported in the literature only in 15 other cases [3]. Solid serous adenoma of the pancreas mimics other pancreatic tumors, especially pNEN, because the stroma demonstrates avid contrast enhancement and accounts for the solid hypervascular appearance on MRI [3]. Few evidence is available in the literature of SSCA mimicking pNEN [3, 6, 8].

Pancreatic cystic neoplasms are mostly benign, and careful observation over surgery is recommended unless the patient manifests symptoms or the tumor begins to show signs of malignancy. However, preoperative diagnosis of SSCA is challenging due to its rarity and similarity in imaging findings to other pancreatic tumors, especially pNEN [3]. A multicenter study by the Japan Pancreatic Society concluded that it is difficult to distinguish SSCA from other pancreatic solid tumors on imaging and even macroscopically at histopathology, since its diagnosis relies solely on its microscopic findings. Surgical resection should be considered only when clear distinction from other pancreatic tumors is difficult, when symptoms or mass effects are present, and when the tumor size is large [9].

Solid serous adenoma of the pancreas typically shows very rich vascularity (especially at the marginal side of the tumor), a faster contrast washout on CT and a signal of very high intensity on T2-weighted MRI, which are not pathognomonic of the disease [3, 10, 11, 15]. Macroscopically, these tumors are well-circumscribed with a solid gross appearance, and complete absence of cystic changes. Microscopically, they are composed of small back-to-back acini with the absence or minute central lumina as shown in our case [7].

Passand et al. [6] reported a 75-year-old woman with a 50-mm solid tumor in the head of the pancreas with partial enhancement on portal phase images on CT, markedly hyperintense features on T2-weighted images on MRI and uptake on 68Ga-DOTATOC PET/CT (SUVmax, 7.2), as for a pNEN. However, histopathology after resection revealed a SSCA. The study suggested that the absorption of 68Ga-DOTATOC on PET/CT should be interpreted with caution, as it does not always indicate the presence of a pancreatic neuroendocrine tumor (pNET) and the possibility of other benign lesions, including SSCA should be considered. Indeed, SSCA expresses somatostatin receptors and therefore can mimic a pNEN.

Knowledge of this disease is important because on imaging its features resemble those of other solid tumors such as pNEN, renal cell carcinoma metastases, and solid pseudopapillary tumor [12]. These types of serous cystadenomas do not contain any cystic spaces on macroscopic histopathology, and their cells are arranged in nests, sheets, and trabeculae separated by thick fibrous bands [13]. In addition, the accuracy of EUS-FNA for diagnosing SCN is only 17% and does not lead to a definitive diagnosis in most patients. The procedure has its limitations, and it alone cannot accurately differentiate SCN from other pancreatic neoplasms [7, 8, 16]. An accurate preoperative diagnosis of SSCA is difficult and remains challenging [3, 6, 8].

Although several guidelines, including those of the European Neuroendocrine Tumor Society (ENETS) [17] and the North American Neuroendocrine Tumor Society (NANETS) [18], recommend preoperative endoscopic ultrasound-guided fine-needle aspiration or biopsy (EUS-FNA/FNB) in cases of suspected pNET, this procedure was not performed in the present case. The decision was based primarily on the imaging findings, which strongly suggested a resectable solid pancreatic neoplasm and the patient's significant abdominal symptoms at the time of evaluation. These clinical factors supported a rapid surgical approach without further delay for histopathological diagnosis. Notably, Kishida et al. [3] also reported a case of SSCA mimicking pNEN, in which EUS-FNA was not performed preoperatively due to diagnostic limitations. Our treatment strategy reflected balanced clinical judgment informed by the patient's clinical presentation on imaging studies and significant symptomatology and literature precedents.

Regarding somatostatin receptor status, we did not perform 68Ga-DOTATOC PET/CT in this case, and we acknowledge this as a limitation of our diagnostic workup. Additionally, immunohistochemical staining for somatostatin receptors was not conducted on the surgical specimen, as the final diagnosis of SSCA was confirmed based on classical histopathological features.

Diagnostic pitfalls in pancreatic cystic neoplasms are well recognized, and even with a complete preoperative workup, including advanced imaging and endoscopic techniques, misdiagnosis still occurs in a significant proportion of cases. Salvia et al. [19] recently reported that at least 20% of patients with pancreatic cystic lesions are misdiagnosed preoperatively, despite adherence to the current guidelines and the use of preoperative endoscopic ultrasound-guided fine-needle aspiration or biopsy. This emphasizes the inherent diagnostic limitations clinicians face in differentiating rare entities such as SSCA from more common neoplasms such as pNEN. Our case further highlights this challenge, reinforcing the need for continued awareness and reporting of atypical presentations.

Due to its rarity, its real incidence and prevalence is unknown. The existing literature consists mainly of individual case reports or small case series. The scarcity of comprehensive statistical information reflects the rarity of the lesion and highlights the need to collect and document specific cases to increase the awareness of the disease.

## 4. Conclusion

We reported a case of a patient with SSCA who was preoperatively diagnosed with a pNEN and underwent laparoscopic pancreaticoduodenectomy. This case reinforces that SSCA is extremely difficult to distinguish preoperatively from other pancreatic solid lesions, particularly pNEN, as its definitive diagnosis relies solely on histopathological findings. Given the rarity of SSCA and its radiologic similarity to other neoplasms, careful consideration of the complete preoperative workup—including functional imaging and tissue sampling—should be emphasized when dealing with presumed rare diagnoses. This approach may help avoid unnecessary extensive surgery in selected cases and highlights the need for continuous awareness of atypical presentations in pancreatic tumors.

## Figures and Tables

**Table 1:** Summary of previously reported SSCA cases.

Author (year)	Country	Age/sex	Tumor size (cm)	Location	Preop diagnosis	Surgery performed
Perez-Ordonez et al. [14] (1996)	Canada	45/F	2.8	Head	pNEN	Whipple
Reese et al. [10] (2006)	USA	50/F	2.5	Body	SPT	DP
Yamaguchi [4] (2006)	Japan	70/M	3.5	Head	pNEN	Whipple
Sanaka et al. [8] (2007)	USA	63/F	4.0	Head	pNEN	Whipple
Stern et al. [12] (2007)	USA	55/F	2.2	Tail	pNEN	DP
Gabata et al. [11] (2005)	Japan	68/M	3.0	Body	SPT	DP
Kimura et al. [9] (2012)	Japan	66/F	3.6	Uncinate	pNEN	Whipple
Kishida et al. [3] (2014)	Japan	67/F	3.5	Body/head	pNEN	Whipple
Passand et al. [6] (2022)	France	75/F	5.0	Head	pNEN	Whipple
Nakamura et al. [7] (2022)	Japan	60/F	2.0	Body	pNEN	DP
Charville and Kao [2] (2018)	USA	58/F	2.9	Tail	SPT	DP
Kim et al. [15] (2008)	South Korea	59/M	2.4	Body	pNEN	DP
Gabata et al. [11] (2005)	Japan	65/F	3.1	Head	pNEN	Whipple
Chu et al. [13] (2017)	USA	62/F	2.7	Body	Metastasis	DP
Belsley et al. [16] (2008)	USA	61/M	2.0	Head	SCN	None

*Note:* pNEN = pancreatic neuroendocrine neoplasm and Whipple = pancreaticoduodenectomy.

Abbreviations: DP = distal pancreatectomy, SCN = serous cystic neoplasm, and SPT = solid pseudopapillary tumor.

## Data Availability

The data that support the findings of this study are available on request from the corresponding author. The data are not publicly available due to privacy or ethical restrictions.
